# Long Non-Coding RNA LINC00152 Regulates Self-Renewal of Leukemia Stem Cells and Induces Chemo-Resistance in Acute Myeloid Leukemia

**DOI:** 10.3389/fonc.2021.694021

**Published:** 2021-07-06

**Authors:** Chunhong Cui, Yan Wang, Wenjie Gong, Haiju He, Hao Zhang, Wei Shi, Hui Wang

**Affiliations:** ^1^ Shanghai University of Medicine and Health Sciences Affiliated Zhoupu Hospital, Shanghai, China; ^2^ Laboratory of Tumor Molecular Biology, School of Basic Medical Sciences, Shanghai University of Medicine and Health Sciences, Shanghai, China; ^3^ Huashan Hospital, Fudan University, Shanghai, China; ^4^ Department of Hematology, First Affiliated Hospital of Soochow University, Suzhou, China

**Keywords:** leukemia stem cells, LINC00152, poly (ADP-ribose) polymerase 1, chemo-resistance, leukemia

## Abstract

Relapse of acute myeloid leukemia (AML) has a very poor prognosis and remains a common cause of treatment failure in patients with this disease. AML relapse is partially driven by the chemoresistant nature of leukemia stem cells (LSCs), which remains poorly understood, and our study aimed at elucidating the underlying mechanism. Accumulating evidences show that long noncoding RNAs (lncRNAs) play a crucial role in AML development. Herein, the lncRNA, LINC00152, was identified to be highly expressed in CD34^+^ LSCs and found to regulate the self-renewal of LSCs derived from AML patients. Importantly, LINC00152 upregulation was correlated with the expression of 16 genes within a 17-gene LSC biomarker panel, which contributed to the accurate prediction of initial therapy resistance in AML. Knockdown of LINC00152 markedly increased the drug sensitivity of leukemia cells. Furthermore, LINC00152 expression was found to be correlated with poly (ADP-ribose) polymerase 1 (PARP1) expression in AML, whereas LINC00152 knockdown significantly decreased the expression of PARP1. Upregulation of LINC00152 or PARP1 was associated with poor prognosis in AML patients. Collectively, these data highlight the importance and contribution of LINC00152 in the regulation of self-renewal and chemoresistance of LSCs in AML.

## Introduction

Acute myeloid leukemia (AML) is a heterogeneous and deadly disease characterized by aberrant myeloid lineage cell proliferation and differentiation (1). Although most AML patients achieve remission, up to 70% of adults and 30% of pediatric patients do not survive beyond 5 years after the initial clinical response due to relapse (2). Historically, the relapse of AML patients has been attributed to the pre-existence of chemoresistant leukemia stem cells (LSCs) (3, 4). Nevertheless, the mechanisms underlying LSC-mediated leukemia relapse have not been elucidated. Leukemia cells differentiate from LSCs (5), which have the capacity to initiate leukemia in immunocompromised mice. Therefore, it is warranted to investigate potential factors contributing to the self-renewal and chemoresistant nature of LSCs.

Long noncoding RNAs (lncRNAs) have been identified in whole transcriptome sequencing projects, such as gene expression profiling interactive analysis (GEPIA). The established functions of lncRNAs include cell cycle regulation, lineage differentiation, and cancer progression (6–8). Deregulated expression of lncRNAs often occurs in AML (9, 10), which has been reported to be independently associated with AML patient prognosis (9, 11, 12). Although lncRNAs have been identified to regulate leukemia progression (13–15), their detailed role in chemoresistance remains unknown. LINC00152 is an lncRNA located at chromosome 2p11.2. Recently, LINC00152 was identified as a potent oncogene in various cancers (16, 17). In particular, LINC00152 expression has been reported to be upregulated in AML samples and facilitate AML leukemogenesis (18); nevertheless, its underlying mechanism needs to be further investigated.

Considering the high expression of LINC00152 in CD34^+^ LSCs and its regulatory role in LSC self-renewal, we hypothesized that LIN00152 could have biological significance in LSC chemoresistance, and the present study was performed with an aim to elucidate this significance. The inhibition of LINC00152 was found to increase the sensitivity of leukemia cells to doxorubicin. Furthermore, LINC00152 expression was correlated with the expression of poly [ADP-ribose] polymerase 1 (PARP1), whereas LINC00152 knockdown markedly decreased PARP1 expression. Thus, our results indicate that LINC00152 may serve as a potential prognostic marker for AML patients.

## Material and Methods

### Cell Culture

Cell lines were purchased from the American Type Culture Collection (Manassas, VA, USA) and were cultured in a humidified incubator at 37°C and 5% CO_2_. 293T cells (ATCC CRL-3216) were cultured in Iscove’s modified Dulbecco’s medium (Thermo Fisher Scientific, Waltham, MA, USA) containing 10% fetal bovine serum (FBS) (Biochrom GmbH, Berlin, Germany) and digested with trypsin-EDTA (Sigma-Aldrich, St. Louis, MO, USA). K562 cells (ATCC CCL-243) were cultured in RPMI-1640 (Sigma-Aldrich) supplemented with 10% FBS (Biochrom GmbH). Cells were grown in log-phase (1 × 10^5^–1 × 10^6^ cells/ml).

### Bone Marrow Cells Isolation

Bone marrow specimens were collected from 15 adult AML patients and analyzed after obtaining written informed consent in accordance with the Declaration of Helsinki.

Bone marrow cells were obtained at the diagnosis of adult patients with AML assessed at the Huashan Hospital of Fudan University, Shanghai, China. Bone marrow samples were collected in 10-ml syringes with 10.000IE/20 ml heparin after bone marrow puncture.

### Flow Cytometry

Bone marrow cells were incubated with Alexa Fluor 488 anti-human CD34 antibody and phycoerythrin anti-human CD38 antibody (both from Biolegend, San Diego, CA, USA) diluted in magnetic-activated cell sorting buffer for 15 min on ice. The staining process was performed under a hood in the dark. After incubation, the cells were washed with phosphate-buffered saline (PBS). Stained cells were kept on ice before sorting. The concentration of the cells was adjusted to approximately 1 × 10^6^ cells/ml. Sorted cells were collected and freshly filtered, with PBS containing 2% FBS used as the catch medium. Acquisition was performed using an LSR II flow cytometer (BD Biosciences, Franklin Lakes, NJ, USA). FlowJo software (BD Biosciences) was used for data analysis.

### Colony Formation Assay

Methylcellulose (Stemcell Technologies, Vancouver, BC, Canada) was placed at room temperature for 30 min before usage. Each selected human LSC was adjusted to a concentration that was 10-fold higher than the final plating concentration. The cell suspension (300 μl) was mixed well with 3-ml methylcellulose at a density of 300 cells per ml of methylcellulose. After removing the air bubbles, the mixture was transferred to a 12-well plate with three replicates for each cell. Colonies (> 50 cells) were stained with trypan blue and counted under a microscope after 10 days.

### Small Hairpin RNA (shRNA) and Plasmid Cloning

LINC00152 shRNAs were cloned into the pLKO.1_GFP vector with *Bam*HI and *Age*I restriction sites. The hairpin was synthesized by Sangon Biotech. The empty vector was recovered after digestion with *Bam*HI and *Age*I. The annealed hairpin and digested vector were ligated with T4 ligase (Thermo Fisher Scientific), and the ligating product was then transformed into *Escherichia coli.* The shRNA-targeting sequences were as follows: forward, CACAGCCGGAATGCAGCTGAA and reverse, CCACTGTGGACTCTGAGGCCT.

### Lentivirus Package and Leukemia Cell Infection

PLKO.1_GFP vector and packaging vectors (PLP1, PLP2, and VSVG) were transfected into 293T cells using Turbofect reagent (Thermo Fisher Scientific). After 48 h, supernatants with lentivirus particles were collected by ultracentrifugation. AML cells were transduced by incubation with the viral particles in the presence of polybrene (8 μg/ml, Sigma-Aldrich). The viral titers were then determined by flow cytometry 48 h following transduction.

### RNA Isolation, Reverse Transcription, and Quantitative Real-Time Polymerase Chain Reaction (qRT-PCR)

AML cells were prepared by centrifugation, and total RNA was isolated using TRIzol reagent (Thermo Fisher Scientific), followed by removal of genomic DNA. Complementary DNA was reverse transcribed using PrimeScript RT Reagent Kit (Thermo Fisher Scientific). qRT-PCR was performed with SYBR Green Master Mix (Bio-Rad, Hercules, CA, USA) on a StepOnePlus Real-Time PCR system (Thermo Fisher Scientific). The primers used for targeting *LINC00152* were (forward) TGGCACAGTCTTTTCTCTACTCA and (reverse) TCAAGAGGTTTCCAGGGGCT, whereas for *PARP1* they were (forward) ACTGACATAGAGAAAAGGCTGGAG and (reverse) GGGGAAACCAGTAAGGCAGAC.

### Drug Sensitivity Assay

K562 cells (control and LINC00152 shRNA) were seeded onto 24-well plates at a density of 2.5 × 10^4^ viable cells/well in triplicate and treated with doxorubicin at the indicated concentrations. Cells were counted and analyzed after 3 days using a cell counting plate under a microscope.

### Statistical Analyses

Statistical analyses were performed using SPSS version 22 (IBM Corp., Armonk, NY, USA) and R studio 3.5.0 (R Foundation, Vienna, Austria). Student’s *t*-test or one-way analysis of variance (ANOVA) was used to identify statistical significance. For Kaplan–Meier survival analysis, the log-rank test was used to determine statistical significance. Statistical significance was set at *P* < 0.05.

## Results

### LINC00152 Is Highly Expressed in LSCs and Correlated With Poor AML Patient Prognosis

**Figure 1:**
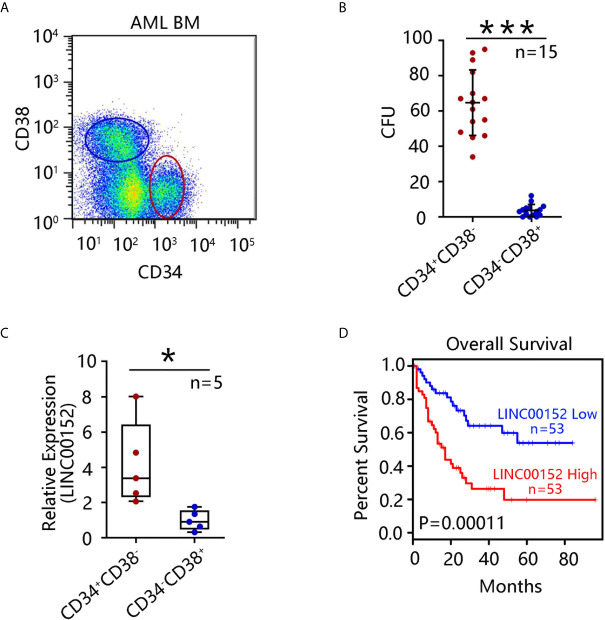
LINC00152 expression is upregulated in leukemia stem cells (LSCs). **(A)** CD34^+^CD38^−^ and CD34^−^CD38^+^ cell subpopulations were sorted by flow cytometry from 15 acute myeloid leukemia (AML) patients. **(B)** Colony formation assay using sorted CD34^+^CD38^−^ and CD34^−^CD38^+^ cells was performed and the mean number of clone forming units was analyzed. Data are presented as the mean ± SEM of three independent experiments (***P < 0.001). **(C)** *LINC00152* expression in CD34^+^CD38^−^ and CD34^−^CD38^+^ cells derived from five AML patients. Data are represented as the mean ± SD of three independent experiments (*P < 0.05). **(D)** Correlation between overall survival of AML patients and *LINC00152* expression (based on a publicly available data set by gene expression profiling interactive analysis (***P < 0.001).

Evidence from a previous study suggests that CD34^+^CD38^−^ stem cells are the leading cause of chemoresistance (19). To investigate the role of this stem cell population in AML, bone marrow cells from 15 paired AML patients were collected, and the CD34^+^CD38^−^ and CD34^−^CD38^+^ subpopulations were sorted using flow cytometry (**Figure 1A**). Notably, CD34^+^CD38^−^ cells possessed a markedly stronger capacity for colony formation than CD34^−^CD38^+^ cells derived from paired 15 AML patients (*P* = 5.4 × 10^−13^) (**Figure 1B**). qRT-PCR showed that *LINC00152* was highly expressed in CD34^+^CD38^−^ cells compared with CD34^−^CD38^+^ subpopulations (*P* = 0.02) (**Figure 1C**). Furthermore, GEPIA, based on The Cancer Genome Atlas (TCGA) data, was performed to determine the functions of LINC00152. Overall, high expression of *LINC00152* was associated with poor AML patient survival (**Figure 1D**), suggesting that LINC00152 may be a potential prognostic marker for AML.

### Correlation Between LINC00152 and LSC-Associated Gene Expression

**Figure 2:**
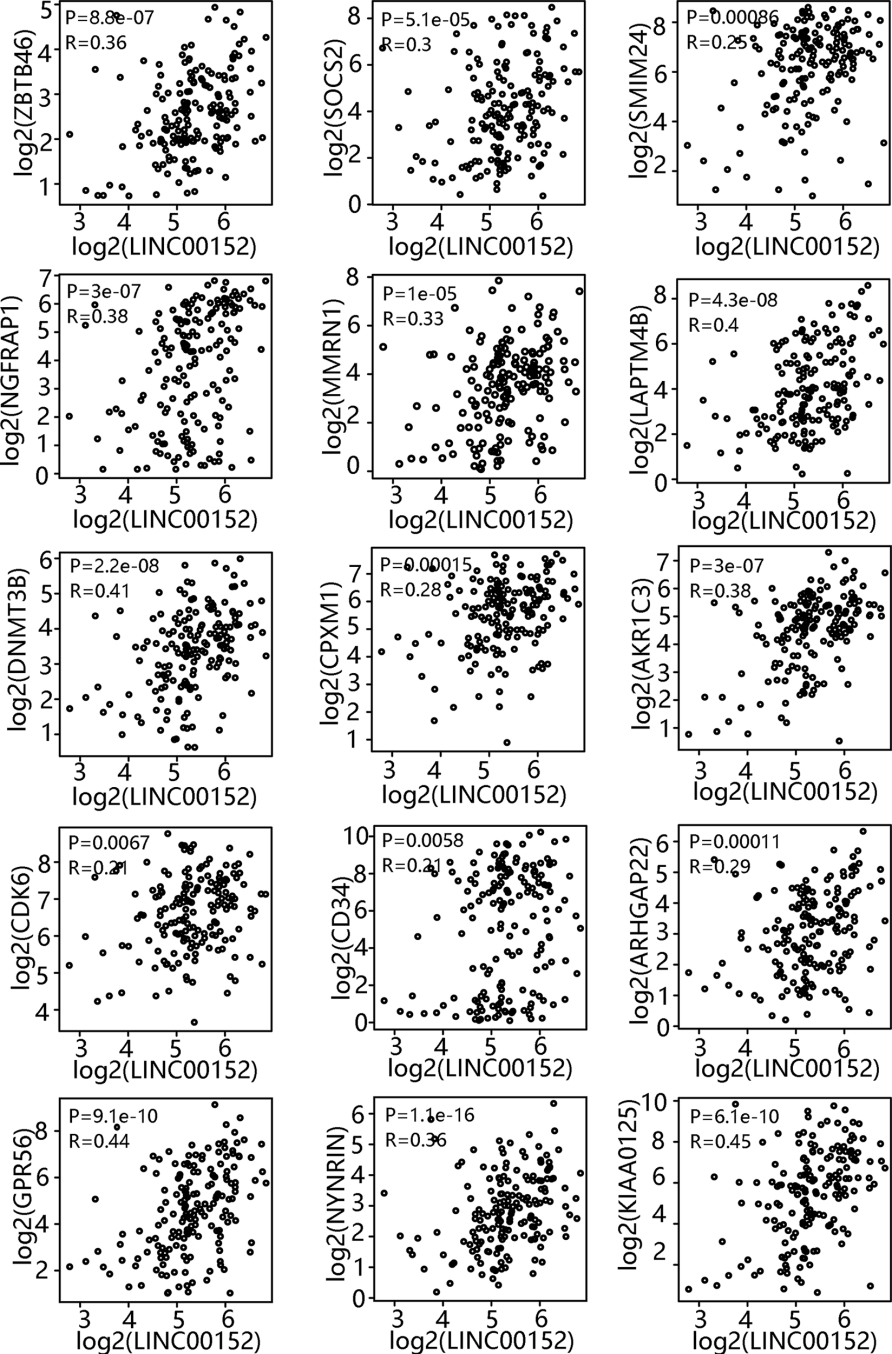
Correlation between LINC00152 expression and leukemia stem cell (LSC)-associated gene expression. LSC17 biomarker gene profile and *LINC00152* expression were analyzed based on a publicly available data set (GSE76009) by gene expression profiling interactive analysis.

Comprehensive analysis of LSC gene expression signatures was previously performed in 78 AML patients and validated by xenotransplantation, which generated a 17-gene LSC (LSC17) biomarker panel (*ZBTB46*, *SOCS2*, *SMIM24*, *NGFRAP1*, *MMRN1*, *LAPTM4B*, *DNMT3B*, *CPXM1*, *AKR1C3*, *CDK6*, *CD34*, *ARHGAP22*, *GPR56*, *NYNR1N*, *KIAA0125*, *EMP1*, and *DPYSL3*). Moreover, Ng *et al.* reported that patients with high LSC17 scores had poor prognosis with current treatments, including allogeneic stem cell transplantation (20). To test whether *LINC00152* expression correlates with the LSC17 gene signature, GEPIA was performed, which revealed that the expression of 15 of the 17 genes was significantly associated with that of *LINC00152* (**Figure 2**). Two uncorrelated genes (*EMP1*, *P* = 0.6; and *DPYSL3*, *P* = 0.69) are not shown. These results suggest an important role of LINC00152 in leukemic stemness.

### Repression of Colony Formation by LINC00152 Knockdown In CD34^+^ AML Cells

**Figure 3:**
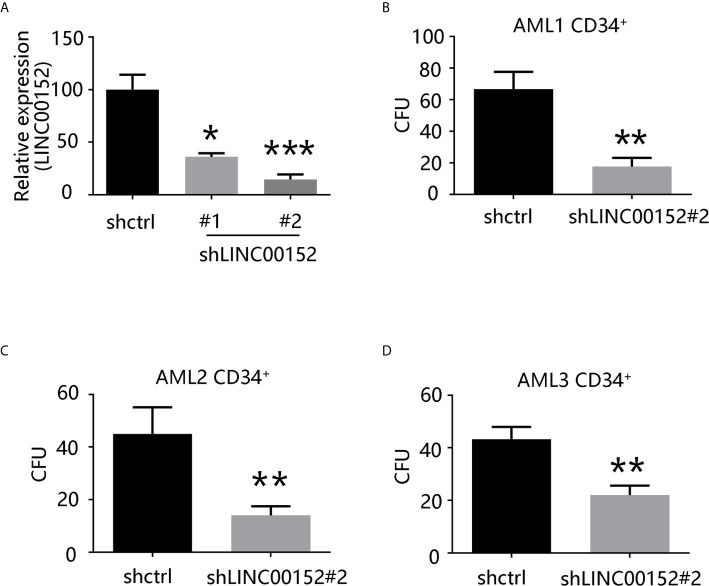
LINC00152 knockdown decreases the capacity of colony formation of LSCs. **(A)** *LINC00152* expression in cells transfected with short hairpin RNAs (shCTRL or sh00152s) (*P < 0.05, ***P < 0.001). **(B–D)** Quantification of colony formation derived from three acute myeloid leukemia patients. Data are presented as the mean ± SEM of three independent experiments (*P < 0.05, **P < 0.01).

*LINC00152* upregulation in LSCs and the observed correlation between LINC00152 and the LSC17 biomarker profile suggested its possible biological contribution toward the stemness of LSCs. To explore the influence of LINC00152 on LSC stemness, knockdown of LINC00152 using targeted shRNAs (sh00152#1 and sh00152#2) was performed. qRT-PCR analysis confirmed that both shRNAs significantly decreased the expression of *LINC00152* compared with control non-targeted shRNA (**Figure 3A**). Colony formation assay is a well-characterized and validated method to assess the differentiation and proliferation ability of primitive hematopoietic cells. Herein, this assay was performed with cells lacking LINC00152. Knockdown of *LINC00152* led to a significant decrease in the colony formation capacity of CD34^+^ cells derived from three AML patients (**Figures 3B–D**), indicating that LINC00152 regulates the self-renewal of LSCs.

### LINC00152 Regulates LSC Chemoresistance Via PARP1

**Figure 4:**
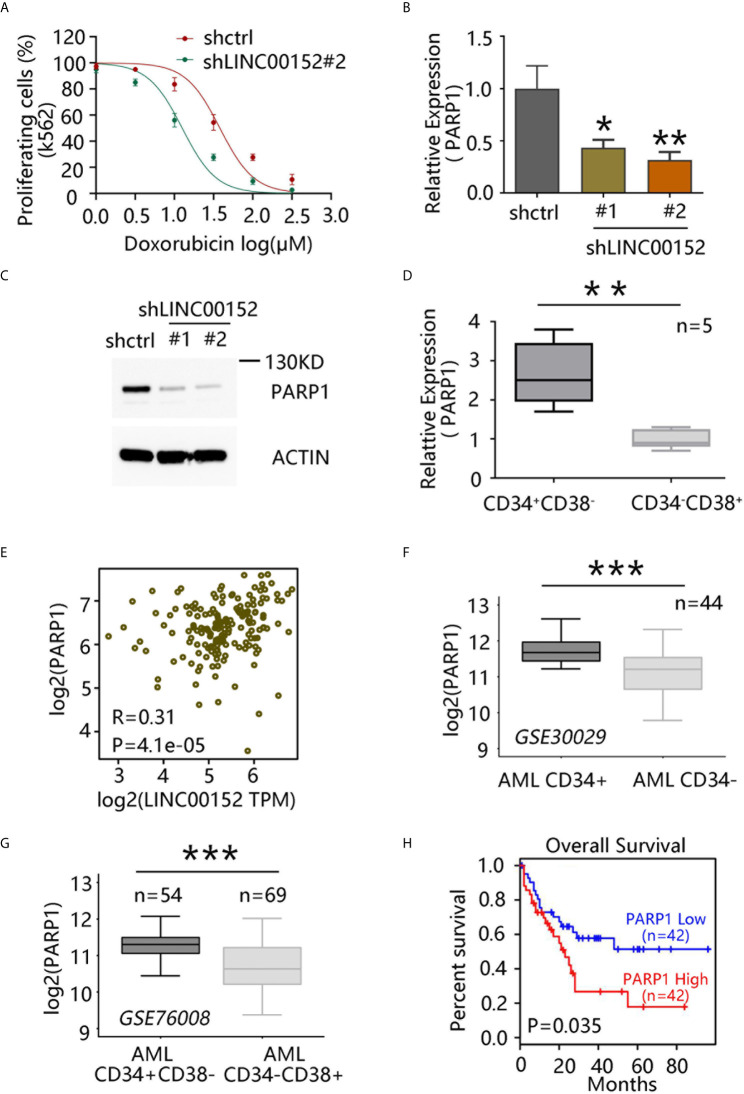
LINC00152 regulates chemoresistance of leukemia stem cells (LSCs) *via* PARP1. **(A)** Doxorubicin sensitivity assay after *LINC00152* knockdown in K562 cells. **(B)** PARP1 mRNA level after *LINC00152* knockdown compared with control acute myeloid leukemia (AML) cells (*P < 0.05, **P < 0.01). **(C)** PARP1 expression at the protein level after *LINC00152* knockdown compared with control AML cells. **(D)** *PARP1* expression in CD34^+^CD38^−^ and CD34^−^CD38^+^ cells derived from five AML patients. Data are represented as the mean ± SD of three independent experiments (**P < 0.01). **(E)** Expression of *LINC00152* was correlated with that of *PARP1* in AML based on a publicly available data set by gene expression profiling interactive analysis. **(F)** *PARP1* expression in paired CD34^+^ cells vs. CD34^−^ cells (GSE30029, ***P < 0.001). **(G)** *PARP1* expression in CD34^+^CD38^−^ vs. CD34^−^CD38^+^ cells (GSE760008, ***P < 0.001). **(H)** Correlation between overall survival of AML patients and *PARP1* expression based on a publicly available data set (GSE76009) by gene expression profiling interactive analysis.

Chemotherapy based on doxorubicin, an anthracycline, remains the standard line of treatment for leukemia (21). As previously described, chemoresistant LSCs partially induce leukemia relapse (3, 4). Whether LINC00152 can regulate the chemoresistance of LSCs to doxorubicin was next investigated. The drug sensitivity curve indicated that *LINC00152* knockdown markedly reduced the chemoresistance of K562 cells to doxorubicin (**Figure 4A**).

PARP1 has been reported to play an important role in DNA damage repair, and its expression is correlated with poor prognosis in AML (22). Moreover, PARP1 inhibition has exhibited the potential to enhance immunotherapy efficacy in AML (23). Thus, whether LINC00152 can regulate the chemoresistance of LSCs through PARP1 was investigated. qRT-PCR analysis confirmed that *PARP1* expression in AML cells was significantly decreased upon *LINC00152* knockdown (**Figures 4B, C** **)**. Moreover, *PARP1* was found to be highly expressed in CD34^+^CD38^−^ subpopulation compared with CD34^−^CD38^+^ cells derived from five AML patients (*P* = 0.0024) (**Figure 4D**). GEPIA further showed that the expression of *LINC00152* was highly correlated with that of *PARP1* (**Figure 4E**). Publicly available microarray data sets were also used to compare *PARP1* expression between CD34^+^ and CD34^−^ cells in AML patients. Overall, *PARP1* was found to be highly expressed in CD34^+^ cells (paired CD34^+^ vs. CD34^−^ subpopulations from 44 AML patients; GSE30029) (**Figure 4F**), as well as in CD34^+^CD38^−^ cells, compared with CD34^−^CD38^+^ cells (CD34^−^CD38^+^ subpopulations from 54 AML patients vs. CD34^−^CD38^+^ cells from 69 AML patients; GSE76008) (**Figure 4G**). Accordingly, PARP1 overexpression was associated with poor prognosis in AML patients (**Figure 4H**). Altogether, these data suggest that LINC00152 modulates the chemoresistance of AML through PARP1.

**Figure 5:**
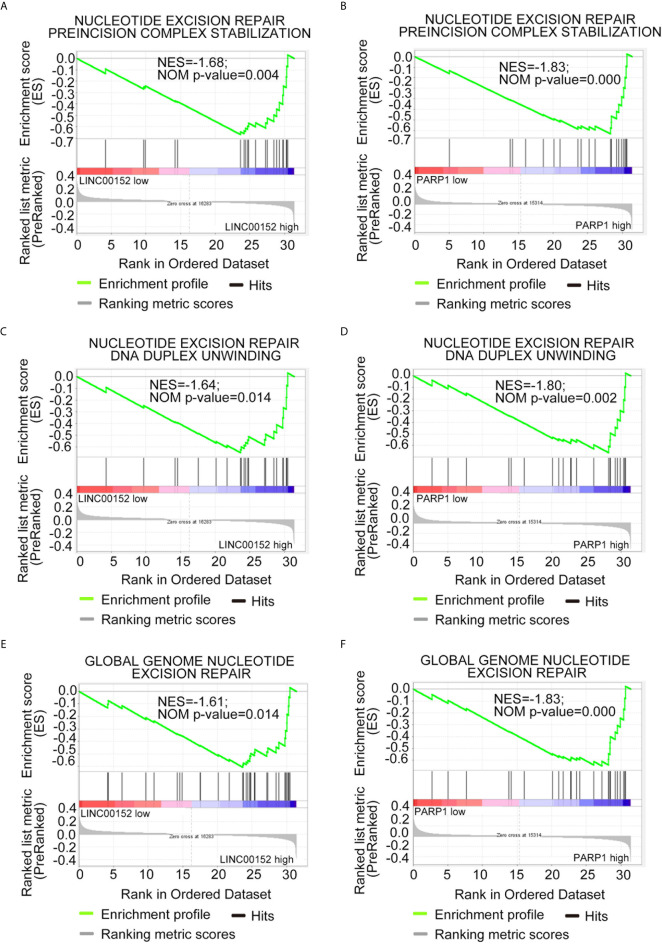
Both *LINC00152* and *PARP1* expression correlated with DNA damage repair in acute myeloid leukemia. High expression of *LINC00152* and *PARP1* individually enriched the genes associated with nucleotide-excision repair, preincision complex stabilization **(A, B)**, nucleotide-excision repair, DNA duplex unwinding **(C, D)**, and global genome nucleotide-excision repair **(E, F)**.

To further explore the potential mechanism through which LINC00152 and PARP1 modulate the chemoresistance of LSCs, Gene Ontology (GO) analysis *via* gene set enrichment analysis was performed. The results of GO analysis showed that both LINC00152 and PARP1 were enriched in processes associated with DNA damage repair-related signaling, such as nucleotide-excision repair preincision complex stabilization, nucleotide-excision repair, DNA duplex unwinding, and global genome nucleotide-excision repair (GSE76009) (**Figures 5A–F** and **Table 1**).

**Table 1:** Gene Ontology analysis.

Gene sets enriched in phenotype (LINC00152)	P-value	Gene sets enriched in phenotype (PARP1)	P-value
NUCLEOTIDE EXCISION REPAIR PREINCISION COMPLEX ASSEMBLY	0.004	SIGNAL TRANSDUCTION INVOLVED IN REGULATION OF GENE EXPRESSION	0.004
POSITIVE REGULATION OF PROTEIN DEPOLYMERIZATION	0.002	GLOBAL GENOME NUCLEOTIDE EXCISION REPAIR	0.000
TRANSCRIPTION COUPLED NUCLEOTIDE EXCISION REPAIR	0.006	NUCLEOTIDE EXCISION REPAIR PREINCISION COMPLEX STABILIZATION	0.000
NUCLEOTIDE EXCISION REPAIR PREINCISION COMPLEX STABILIZATION	0.004	RRNA METABOLIC PROCESS	0.000
NUCLEOTIDE EXCISION REPAIR DNA INCISION	0.008	RRNA MODIFICATION	0.000
NUCLEOTIDE SUGAR METABOLIC PROCESS	0.000	EXTABLISHMENT OF PROTEIN LOCALIZATION TO TELOMERE	0.000
INTRACILIARY TRANSPORT	0.010	RIBOSOME BIOGENESIS	0.000
REGULATION OF HEMATOPOIETIC PROGENITOR CELL DIFFERENTIATION	0.000	DNA DAMAGE RESPONSE DETECTION OF DNA DAMAGE	0.000
NON RECOMBINATIONAL REPAIR	0.004	NUCLEOTIDE EXCISION REPAIR DNA DUPLEX UNWINDING	0.002
ERROR FREE TRANSLESION SYNTHESIS	0.012	TRANSLATIONAL TERMINATION	0.006
OLIGOSACCHARIDE LIPID INTERMEDIATE BIOSYNTHETIC PROCESS	0.008	MITOCHONDRIAL TRANSLATION	0.004
NUCLEOTIDE EXCISION REPAIR DNA DUPLEX UNWINDING	0.014	NCRNA PROCESSING	0.000
PURINE NUCLEOSIDE MONOPHOSPHATE METABOLIC PROCESS	0.010	GLUTAMINE METABOLIC PROCESS	0.000
HISTONE UBIQUITINATION	0.006	AMINO ACID ACTIVATION	0.006
RNA 3 END PROCESSING	0.006	NCRNA METABOLIC PROCESS	0.000
G0 TO G1 TRANSITION	0.010	PURINE NUCLEOSIDE MONOPHOSPHATE BIOSYNTHETIC PROCESS	0.000
REGULATION OF SIGNAL TRANSDUCTION BY P53 CLASS MEDIATOR	0.000	POST ANAL TAIL MORPHOGENESIS	0.004
HEMATOPOIETIC STEM CELL DIFFERENTIATION	0.004	MATURATION OF SSU RRNA FROM TRICISTRONIC RRNA TRANSCRIPT SSU RRNA 5.8S RNA LSU RRNA	0.004
MRNA 3 END PROCESSING	0.006	NEGATIVE REGULATION OF TELOMERE MAINTENANCE	0.004
GLOBAL GENOME NUCLEOTIDE EXCISION REPAIR	0.014	RIBONUCLEOPROTEIN COMPLEX BIOGENESIS	0.004

The highlights mean the same signalling enriched in LINC00152 and PARP1.

## Discussion

LSCs are the most important contributors toward drug resistance and disease relapse in AML, with the proportion of LSCs in patients with AML being closely related to poor prognosis (20). A better understanding of the regulatory mechanisms of LSCs is necessary to effectively combat AML. In recent years, lncRNAs have been reported to play a key role in the growth and differentiation of cancer stem cells, in addition to being involved in cancer metastasis and drug resistance development (24, 25). Hence, the field of lncRNAs has attracted increasing attention, with drug development targeting lncRNAs emerging as a new direction. Theoretically, targeting lncRNAs can be achieved through blocking or knockdown by either antisense oligonucleotides (ASOs) or small interfering RNAs (siRNAs). However, the specific transport of ASOs or siRNAs to target cells and ensuring their stability remain considerably challenging (26).

In this study, it was confirmed that CD34^+^CD38^−^ cells from AML patients possess strong LSC characteristics and tumor formation potential. Moreover, high expression of *LINC00152* was found to be significantly correlated with the poor prognosis of AML patients. Indeed, *LINC00152* expression was significantly correlated with that of 15 genes within 17 biomarker genes, which could accurately determine the drug resistance of AML patients. Furthermore, the present experimental data revealed that *LINC00152* expression was significantly increased in CD34^+^CD38^−^ cells and that its knockdown significantly reduced the colony formation ability of LSCs obtained from three AML patients. Taken together, these data indicate that the self-renewal ability of LSCs is greatly modulated by LINC00152, but the underlying mechanism requires further clarification.

LINC00152 is a large intergenic noncoding RNA with a length of 852 bp, located on chromosome 11.2 of genome 2 (16). Recent studies have shown that LINC00152 is highly expressed in various tumors, such as glioma, non-small cell lung cancer, and gastric cancer. Upregulated LINC00152 interacts with several signaling pathways, thereby promoting inflammatory responses as well as tumor cell proliferation, invasion, and metastasis (27–30). Recent studies have also reported that LINC00152 knockdown suppresses proliferation, accelerates apoptosis, and induces cycle arrest in AML cells (18).

Herein, the expression of *PARP1* was found to be significantly decreased when *LINC00152* was downregulated. PARP1 is mainly involved in DNA damage repair and is overexpressed in cancer stem cells. Previous studies have reported that high PARP1 expression is associated with poor AML prognosis (3) and the treatment with the PARP1 inhibitor enhances anti-AML effects (22). This is consistent with the results presented herein, which demonstrate that *PARP1* is highly expressed in LSCs and that its expression is significantly correlated with that of LINC00152 in AML. Additionally, the findings revealed that high *PARP1* expression was correlated with poor overall survival in AML. Moreover, along with the knockdown of *LINC00152*, *PARP1* expression was significantly decreased, which in turn increased the sensitivity of cancer cells to the DNA damaging agent doxorubicin. Consistently, GO analysis showed that genes of LINC00152 and PARP1 were mainly enriched in processes associated with DNA damage repair-related signaling, such as nucleotide-excision repair, preincision complex stabilization, nucleotide-excision repair, DNA duplex unwinding, and global genome nucleotide-excision repair. Chemotherapy is the standard treatment for AML; however, the prognosis of AML patients who relapse remains poor. Our study will be useful for exploring novel targets to induce DNA damage and perturb cellular DNA damage repair. However, our results contrast with data from breast cancer (31), in which lncRNA H19 overexpression promoted doxorubicin resistance by downregulating PARP1 expression. Therefore, the detailed regulatory network of LINC00152 and PARP1 in AML needs to be further explored.

lncRNAs display significant expression variability and subcellular localization diversity. Accumulating evidence shows that a novel regulatory mechanism exists between lncRNAs and microRNAs. lncRNAs can act as endogenous molecular sponges to compete for the targets of the microRNAs, thereby negatively regulating the expression of microRNAs. For example, Chen et al. reported that the lncRNA UICLM could function as a competing endogenous RNA (ceRNA) for miR-215 in colorectal cancer cells to regulate the expression of ZEB1 (32), and Li et al. demonstrated that lncRNA ZFAS1 functions as an oncogene in hepatocellular carcinoma progression by binding to miR-150 and abrogating its tumor-suppressive function (33). In particular, it has been reported that LINC00152 acts as a ceRNA for miR-185-3p and miR-632 to regulate FSCN1 expression in colorectal cancer (34) cells, and recent studies have reported that ectopically expressed LINC00152 accelerates AML proliferation and targets the miR-193a/CDK9 axis to exert its effect (18). Since PARP1 is a major downstream target of miR-181a (35), LINC00152 may also regulate PARP1 through microRNAs. Furthermore, posttranslational modifications of PARP1, such as mono (ADP-ribosyl)-ation (36, 37), phosphorylation, methylation, and acetylation, can modulate its activity. Thus, LINC00152 may influence PARP1 posttranslational modification by binding to the enhancer of zeste homologue 2 (EZH2) (38) or participate in the phosphatidylinositol 3-kinase/AKT signaling pathway (39), which may also be important for PARP1 expression and activity.

In conclusion, our study shows that *LINC00152* is highly expressed in LSCs and correlated with *PARP1* expression, with the inhibition of LINC00152 leading to the downregulation of *PARP1*. Moreover, expression of LINC00152 or PARP1 is associated with poor prognosis in AML patients. Functionally, it can be speculated that LINC00152 regulates the self-renewal of LSCs and induces chemoresistance *via* PARP1. Therefore, these findings suggest that the LINC00152/PARP1 pathway could serve as a novel therapeutic target for AML.

## Data Availability Statement

The original contributions presented in the study are included in the article/**Supplementary Material**. Further inquiries can be directed to the corresponding authors.

## Ethics Statement

The studies involving human participants were reviewed and approved by Huashan Hospital. The patients/participants provided their written informed consent to participate in this study.

## Author Contributions

CC participated in the concept and design experiments, collect data, analyzed and explained the data, and wrote the manuscript. YW provided the study material. WG provided the study material. HH provided study material. HZ provided study material, concept and design experiments, financial support. WS participated in the concept and design experiments, wrote the manuscript, and made final approval for the manuscript. HW participated in the concept and design experiments, financial support, write manuscript, finally approve manuscript. All authors contributed to the article and approved the submitted version.

## Funding

This work was supported by National Natural Scientific Foundation of China Grants (81802961).

## Conflict of Interest

The authors declare that the research was conducted in the absence of any commercial or financial relationships that could be construed as a potential conflict of interest.
