# Supplementary figures and images for: Long Non-Coding RNA LINC00152 Regulates Self-Renewal of Leukemia Stem Cells and Induces Chemo-Resistance in Acute Myeloid Leukemia
Source: Front Oncol. 2021 Jul 6;11:694021. doi: 10.3389/fonc.2021.694021 (PMC8290167; doi:10.3389/fonc.2021.694021)

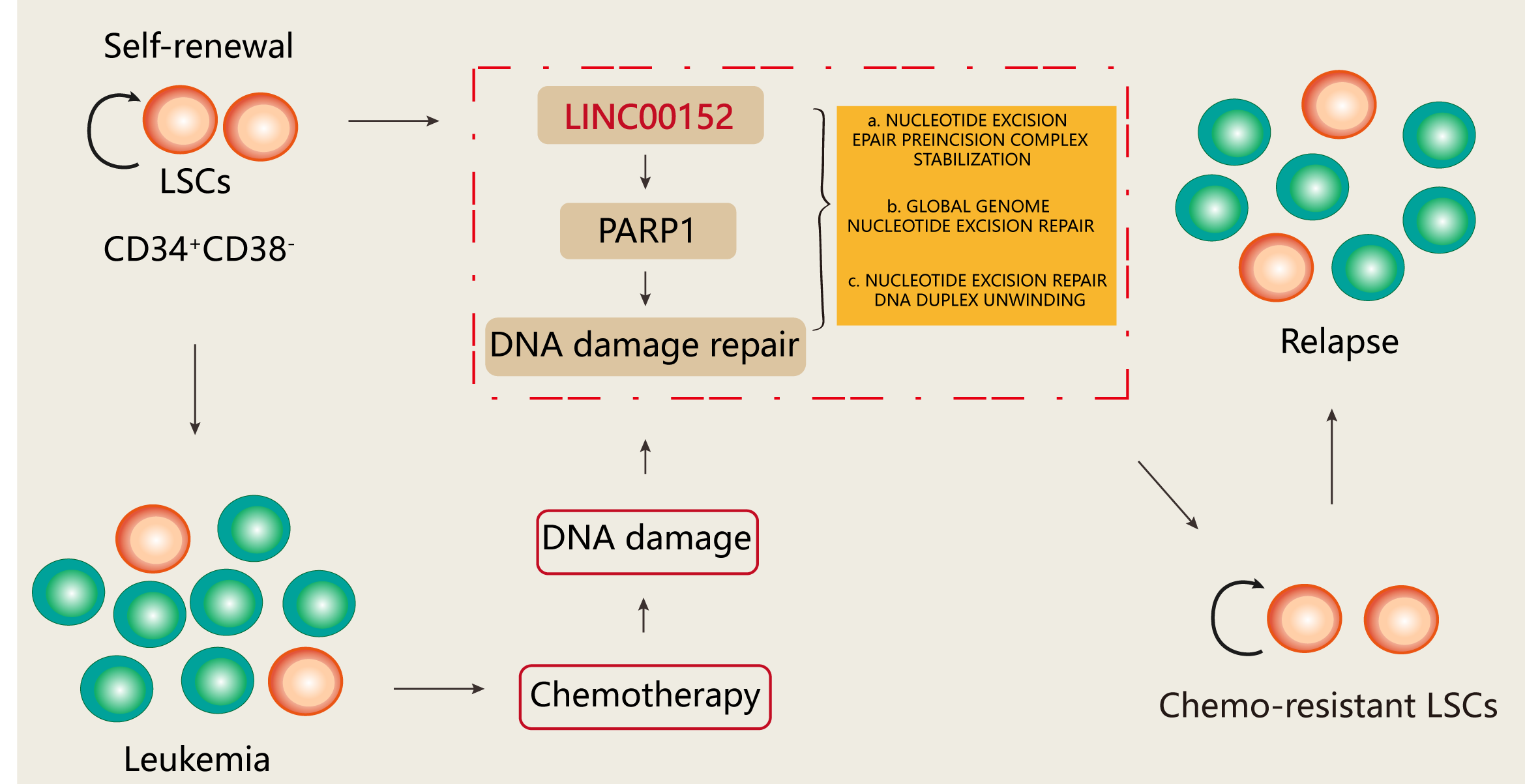

Supplement: Supplementary file 1 [file Image_1.tif]
